# Create to Collaborate: using creative activity and participatory performance in online workshops to build collaborative research relationships

**DOI:** 10.1186/s40900-023-00512-8

**Published:** 2023-12-06

**Authors:** Alice Malpass, Astrid Breel, Jo Stubbs, Tassos Stevens, Persis-Jadé Maravala, Ellie Shipman, Zoe Banks Gross, Michelle Farr

**Affiliations:** 1 The National Institute for Health and Care Research Applied Research Collaboration West (NIHR ARC West) at University Hospitals Bristol and Weston NHS Foundation Trust, Bristol, UK; 2 Population Health Sciences, Bristol Medical School, University of Bristol, Bristol, UK; 3 Research and Graduate Affairs, Bath Spa University, Corsham Court Campus, Bath, SN13 0BZ UK; 4 Elizabeth Blackwell Institute, University of Bristol, Bristol, UK; 5 Coney Ltd, Toynbee Studios, 28 Commercial Street, London, E1 6AB UK; 6 ZU-UK (Zecora Ura Theatre Company Limited by Guarantee), St Mary’s Complex, Waverley Street, Bootle, L20 4AP England; 7 Freelance Artist, Illustrator and Workshop Facilitator, Bristol, UK; 8 Knowle West Media Centre, Leinster Ave, Bristol, BS4 1NL UK; 9 Sustrans, 2 Cathedral Square, Bristol, BS1 5DD UK

**Keywords:** Creative activity, Collaborative research, Creative partnerships, Public engagement, Public involvement, Participatory performance

## Abstract

**Background:**

Creative methods/practices have been highlighted as helpful to develop more collaborative, equitable research partnerships between researchers and communities/public-participants. We asked artist partners to design four online workshops, one on each research priority area: school environments and mental health; wellbeing within the Somali community; air pollution; health data. We aimed to understand whether creative processes can enable public-participants and researcher- participants to meet in a neutral space to discuss a research theme and begin to build collaborative relationships through more equal engagement. Ideas could be taken forwards with seed funding, providing opportunity for collaboration to continue beyond initial workshops.

**Methods:**

Different artist partners designed and facilitated four workshops. Evaluation data was collected on each workshop using participatory observation and fieldnotes, alongside chatlog data, and one-to-one interviews with 21 workshop participants, providing a contextually rich, comparative evaluation across four diverse workshops. Analysis was thematically driven.

**Results:**

Artist partners took different approaches to designing workshops. The workshops began with introductory games and activities, and there was less emphasis on introductions of people’s roles, with the intention to avoid hierarchical dynamics. Whilst public-participants enjoyed this, some researchers found it challenging and reported confusions over their workshop roles. Disrupting usual practice and challenging norms was not always an easy experience. There were examples where emergent, co-created knowledge was enabled. However, it was more challenging to facilitate longer-term collaborative research projects from the workshops due to different stakeholder priorities, and lack of staff time/ less sense of ownership for further work.

**Conclusions:**

Creative activities can influence and impact the types of conversations between public-participants and researchers in a way that changes and challenges power dynamics, shifting towards public-participant driven discussion. Whilst deconstructing hierarchies is important, supporting researchers is key so that any discomfort can be productive and experienced as a vital part of co-production. Longer term collaborative research projects were limited, highlighting a need for facilitation beyond initial workshops, and a sense of ownership from workshop participants to take things forwards. Workshops like these may lend themselves well to research prioritisation. However, taking community-led ideas forwards within research funding landscapes remains challenging.

**Supplementary Information:**

The online version contains supplementary material available at 10.1186/s40900-023-00512-8.

## Abstract (plain language summary)

Create to Collaborate aimed to develop and facilitate creative public involvement workshops with members of the public, researchers, and community organisations, who were potentially interested in collaborating on a future health research project. We wanted to evaluate the extent to which creative, external facilitation by different artists could enable the development of more equal relationships and reduce power differences at the beginning of a research process. We worked with artists who designed and facilitated an online workshop, and community partners who helped recruit members of the public to increase diversity in participation. Then, we delivered and evaluated the workshops to understand different participants’ experiences. Four workshops are reported in this article, each focused on a different topic: mental health in school environments, wellbeing in Bristol’s Somali community, air pollution, and health data. We took observational notes at the workshops and conducted one-to-one qualitative interviews with 21 workshop participants. Feedback from public-participants taking part in creative workshops led by artist facilitators described them as ‘engaging’, ‘comfortable’ and ‘equitable’. Researcher-participants reported workshops as ‘open’, ‘inclusive’ and ‘engaging’, whilst also reporting some confusion over what their own roles were and how much they could contribute as academic researchers. The project demonstrates a need for further clarity, particularly for researchers, over what roles may be in setting up a collaborative relationship, and possible disparity in expectations between those attending in an employment capacity and those joining as a public-participant in their own time.

**Supplementary Information:**

The online version contains supplementary material available at 10.1186/s40900-023-00512-8.

## Background

Co-production research methods facilitate researchers, practitioners and the public to work together, so that they share power and responsibility from the start to the end of the project, including the generation of knowledge [1]. There is a strong theme in the co-production literature that highlights the importance of ensuring that hierarchical structures are interrogated, and levelled where possible, with a growing body of knowledge on how to achieve this and what practical strategies are effective [2, 3]. One area that offers a potential direction for travel is interest in the role of creativity and arts in the co-production of health research and in practices of knowledge mobilisation [4–9].

### Creativity and participatory performance

Create to Collaborate (C2C) was designed to focus on creative participatory processes, exploring creativity as an enabling, dynamic *process*, rather than the development of a creative *product*. Standard definitions of creativity often focus on outcomes rather than process [10]. Research into creativity has more recently highlighted the importance of paying attention to the dynamic process of creativity that may or may not lead to creation [10]. The identification and conceptualisation of an unsolved problem can be considered to be a creative task in itself [10]. More recently, work to define a “creative experience”, has been proposed as follows:

*“A creative experience can be defined as novel person world encounters grounded in meaningful actions and interactions, which are marked by the principles of: open-endedness, nonlinearity, pluri-perspectives and future-orientation”* [11].

Knowledge production environments such as Universities often privilege rationality and reason, rather than emotion, expression or creativity [12]. By working with artists we intended to explore approaches to participation that moved away from rational discourse and traditional meeting structures into differently structured and unexpected encounters. We intended to explore creative-based research as a process, rather than a product to disseminate results [4].

Specifically, we were interested in how participatory performance [13, 14] and other creative processes might contribute to meaningful experiences of participation within the development of health research. Participatory performance (as a specific set of performance practices) contains significant expertise around how to design experiences for participants that are meaningful, facilitated appropriately, and achieve the desired type of engagement with the subject of the performance [14, 15]. The skills that underpin this ability to successfully design an experience for participants include knowing how to invite participation, how to facilitate and incorporate the contributions made by participants, and the ability to build interpersonal relationships between the participants as well as between participant and performer [14, 15]. Participatory performance makers also work thoughtfully with the power differentials at play in different participatory situations; appreciating how difficult it is to achieve genuine collaboration with audiences [15]. Offering the ability to contribute may not always result in participants experiencing agency in that situation, and agency needs to be experienced in order to be meaningful [16]. Public involvement and engagement in health research can be considered as a specific type of participatory situation, which requires similar components as a performance to be successful: a clear invitation for participants, a process through which their contributions can become part of the project, and the facilitation of relationships between all participants. We wanted to explore how the expertise of participatory performance makers and artists might be productively applied within a public involvement with research context.

### Aims and research questions

The aim of this project was to explore how creative engagement methods might enable different sectors of the public who might not usually get involved within research projects, to participate in the forming of research agendas and projects, and support the development of more equal relationships. In this article we explore how creative processes and participatory performance influence the relational dynamics between the public and researchers within the four workshops held to answer three research questions:

1. How can creative activities facilitate the development of more equal or neutral interpersonal relationships between researchers, practitioners and public-participants, and begin to support the active deconstruction of power hierarchies present in engaged and collaborative research?
2. How do creative activities impact on perceived roles and opportunities for contribution in collaborative health research projects (i.e. potential agency and/or increased capacity to engage)?
3. What is the impact of these creative engagement methods on:
  - the potential collaborative health research projects
  - longer-term relationships between researchers, practitioners and public-participants?

This paper tackles the first two research questions, and we illustrate our steps and recommendations towards the third.

## Methods

### Study design

This qualitative study involved two interrelated phases.

**Phase 1:** identifying and working with:

- Artist partners who could design, deliver and facilitate an online public involvement workshop with a creative aspect (designing the workshop)
- Community partners to support recruitment of members of the public who are less represented within research projects, and who may experience barriers to getting involved in health research (with the aim of increasing diversity in research participation).

**Phase 2**: evaluating the C2C public involvement workshops through a qualitative study to understand whether and how creative engagement methods and participatory performance can facilitate the development of more equal or neutral interpersonal relationships between researchers, practitioners, and public-participants.

The core C2C team involved two health researchers (a health sociologist (MF) and anthropologist (AM)), a public engagement expert (JS), and a participatory performance and impact researcher (AB). The C2C project involved many groups, including both those who delivered and participated in the project. See Table 1 for a full overview of all groups involved in the project and how we refer to them through this article.

**Table 1:** People involved in the C2C project with terms for reference

	Name of group involved	Role in project	Relationship to research institute
Delivered project	C2C team	Core project team, made up of 3 researchers (MF, AM, AB) and 1 public engagement expert (JS), responsible for the overall delivery and evaluation of project	Internal
	C2C team observer	Member of C2C team making observational notes on workshops, for data collection (AM for all workshops plus one other)	Internal
	Artist partner	External collaborators; artists responsible for the design and facilitation of project workshops	External
	Community partner	Local community organisations who supported workshop design and participant recruitment e.g. young people’s mental health charity, community arts centre	External
Participated in project	Public-participant	A member of the public/community who attended and took part in workshop	External
	Researcher-participant	An academic researcher from the University of Bristol who attended and took part in workshop	Internal
	Practitioner-participant	An individual from a community organisation who works in an area related to workshop theme, who attended and took part in workshop (e.g. health link worker)	External

### Phase 1: working with artist and community partners

C2C was originally conceived and initiated before the COVID-19 pandemic as a series of face-to-face workshops combining a morning creative activity, shared lunch and some research focussed discussions in the afternoon. After an initial face-to-face pilot with young people, we had to transform all our plans into a digital online format as the pandemic started and lockdowns occurred. This meant new challenges for how we could bring the creative element online, and we shortened our proposed structure from five hours to 90–180 min with breaks.

Participatory performance has a history of using different types of technology to mediate participation [17, 18], so when our workshops had to take place online, we built on existing expertise to develop online workshops. We worked with artist partners to rework our original ideas and explore how we might achieve the same aims online, but without just trying to recreate what we had originally planned in a face to face format before the pandemic. This included taking a wider view on what a shared creative activity might look like: in person this might have been all making a pot out of clay together but online this would not create connections between participants as each would be working individually in their own space. Two C2C researchers (AM and AB) had informal conversations with an agreed shortlist of potential artist partners. Our criteria for choosing artist partners included experience and knowledge of facilitating online creative events, knowledge and experience of participatory performance and/ or other creative participatory processes, expertise in working with community groups (that related to our chosen participant groups) and a range of creative approaches.

The C2C team agreed upon three artist partners who were approached and asked to design a workshop (WS) on at least one of the four research priority topic areas: school environments and mental health (WS1), wellbeing within Bristol’s Somali community (WS2), air pollution (WS3), and health data (WS4). Each of the four workshops was designed bespoke to the topic and the people we were hoping to involve. Workshop topic areas were chosen through consideration of institutional priorities, in discussion with various academic, community organisation and public contributor stakeholders. Two were set within the research themes of the Elizabeth Blackwell Institute (EBI), a University of Bristol hub for interdisciplinary health research, and two were set within the context of the NIHR ARC West research priority setting exercises (school environments and mental health, air pollution). NIHR ARC West conducts applied health research with partners in the health and care sector, alongside patients and members of the public to address issues facing the health and social care system.

### Rationales for workshop design

One key challenge within collaborative development of research projects is that it is difficult to manage power differentials when researchers know more about how a research project might happen than potential participants. Often public involvement workshops are designed by the researchers with their own aims and ambitions in mind [19]. We aimed to change some of these dynamics within the workshop design and our rationales are outlined in Table 2.

**Table 2:** Rationales for workshop design

Aims/ actions	Rationale
Artists designing the workshop	Employ skilled facilitators with appropriate skill sets that were external to the C2C team and the University, to support the facilitation of a more neutral space [7] Provide space for artists and facilitators to design a situation that builds on their expertise, without the limitations that can happen when academics commission an artist and then direct them (but without having any experience design expertise themselves). Academics relinquish control [5] Avoid academics setting up the workshop, where they might make implicit decisions about what is important and what should be discussed, and they would know exactly what would happen and when Create a space where it might become possible for the facilitator to subvert what the participants’ expectations might be for their role and the purpose of their contributions
Creating a level playing field at the beginning	Researcher-participants enter the workshop space with the same level of knowledge as the public-participants and having been told the same reasons for why they might want to attend. We note that whilst the C2C team aimed for researcher-participants to be taken through the same recruitment process as public-participants, in practice researcher-participants would never enter a research project as a 'naïve participant' as they already have a deep understanding of research processes (e.g. consent procedures and investment in topic areas through their own research careers) Destabilise hierarchy [5]
Change the usual introductions style	Introducing a researcher-participant at the start suggests a structure of a focus group, where the researcher has expertise in a particular topic and where they would like to collect the experiences of people who have lived experience of that topic. Without introducing the researcher-participants, we intended to create a more open space for participants to consider their own role within the event and begin at a more level starting point In most workshops we intended that researcher-participants were not introduced as a researcher, but rather only by name in the same way as public-participants. However in some workshops this was less feasible (in WS1 the public-participants were young people, making the adults in the workshop clearly researcher-participants, in WS3 one researcher was known to the group.)
First build interpersonal connections, before discussing research interests	Use a variety of techniques including participatory performance, creative methods and play to build interpersonal connections between people first, before then beginning a conversation about their shared interests and respective experiences and ideas on the research topic Actively cultivate relationships as a priority [5]
Final criteria for workshop design	Build relationships between public-participants and researcher-participants in a way that attempts to break down common hierarchies in research Include a section that is ‘for fun’ rather than about research; focused on building relationships where everyone works together doing something fun/creative Include a section focused on collective development of research ideas in relation to the workshop theme, using creative methods and including collaborative processes of capturing conversation and outcomes
Workshop design lead	Artist partners and community partners, together with AB and JS (WS1-2, WS4) Artist partner and community partners together with JS, AM and MF with input from topic researcher (WS3)

### Recruitment and informed consent

**Fig. 1:**
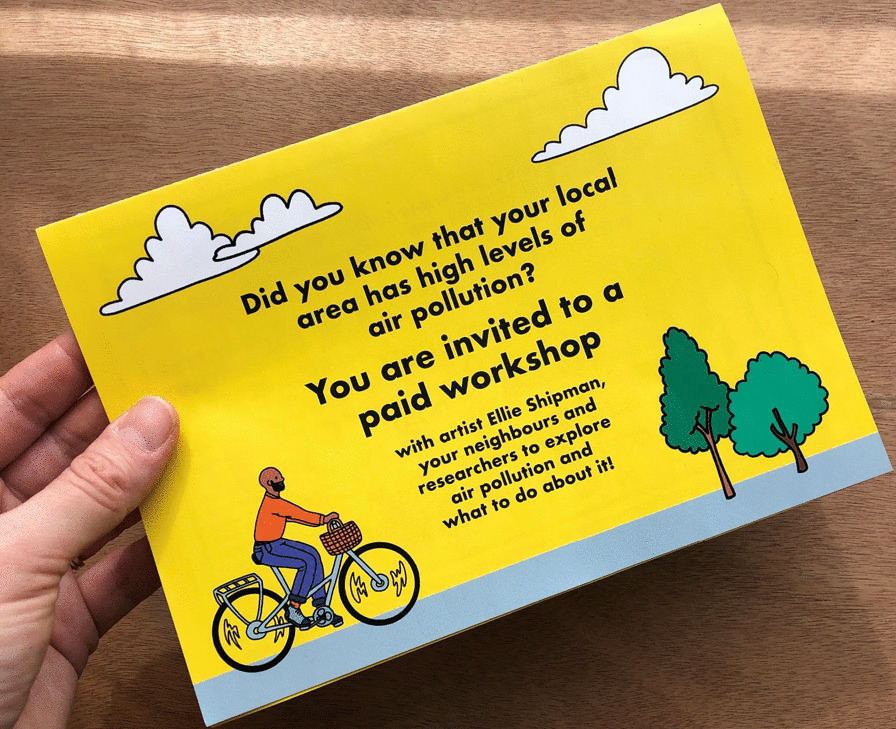
Workshop 3 Air pollution leaflet using a physical recruitment strategy due to geographical recruitment. Other workshops used digital recruitment

We identified community partners with the appropriate expertise and established community links for three of the four workshop priority topic areas (school environments and mental health, wellbeing within the Somali community, air pollution). Community partners were seen as the most appropriate partners who had specialism in the area, often had links to community participants and could advertise workshop invitations to reach people who were less represented within research projects, and who may experience barriers to getting involved. Community partners promoted the invitations through various means including a shared survey link on their website, social media, newsletters, and email listings for potential workshop participants to click and sign up. With WS3 we advertised and recruited through leafleting in areas of deprivation with high levels of air pollution (see Fig. 1 for the leaflet) and did a more open call out for participants for WS4 through creative organisations. The survey link gathered information on eligibility criteria which was different for each workshop (see Table 3). As workshops progressed we added an additional question in the sign-up survey as to whether people had been previously involved with research, positively sampling those who had been less involved in research.

**Table 3:** Workshop theme, design strategies, recruitment mechanisms and inclusion criteria

Invitations & recruitment	Workshop 1: Mental health and school environments	Workshop 2: Wellbeing in Somali community	Workshop 3: Air pollution	Workshop 4: Health data
How the workshop theme was developed	Young people’s advisory group input into ARC West research priority setting processes	Discussion between C2C project staff and community organisation	Discussion with ARC West public health research priority setting group	Discussion between C2C staff and researchers working on health data projects across multiple disciplines
Artists’ workshop design strategy used for facilitated co-creation	–Instructions and playful tasks as Rancièrian pedagogical tools, facilitating democratic knowledge exchange and production - Games as social structures generating temporary communities - Playfulness and silliness allowing for norm-shedding and intimate connections between strangers – Foregrounding positionality and context-specificity – Co-designed and co-facilitated by Persis-Jadé Maravala and Hayley Dawn Hill, ZU-UK	– It was important to bridge to the participating Somali women’s community, so selected a female-identifying team including Yusra Warsama, a freelance maker from Coney’s Guild, herself with Somali family heritage - The workshop was a hybrid of typically Coney pieces of resonant gameplay plus reflection, with Yusra’s own creative writing practice – Co-facilitated by Eliza Cass & Yusra Warsama, co-designed with Tassos Stevens, Coney	– To enable the online workshop to be as accessible, interactive and inclusive as possible, physical flyers were created to advertise it, and an illustrated paper-based workshop pack was developed for participants to fill in the exercises live, directly responding to the hosted session. This was posted in advance to participants – Co-designed and co-facilitated by Ellie Shipman & Zoe Banks Gross	– We presented a contest between teams, curated to mix researchers and public, with a light fiction contextualising the research themes – The contest comprised resonant pieces of gameplay plus reflection, early rounds bonding teams and priming themes, culminating in co-design of research framed as the final challenge – Co-designed and co-facilitated by Rhianna Ilube & Tassos Stevens, Coney
Invitations & recruitment mechanisms for public-participants	Young people’s mental health charity, social media, websites	Local community organisation and associated network links	Leafleting through doors in neighbourhoods with high air pollution and high indices of multiple deprivation. Social media adverts, emails to local community groups	Creative organisations mailing lists both in Bristol and nationally
Inclusion criteria for public-participants	Aged 12 to 18 Attending school Speak English	Over 18 Part of Somali community and/or works with Somali community Speak English Identifies as female	Over 18 Resident in designated area of high pollution/based on postcode/ street LA data Speak English	Over 18 Based in the UK at time of the workshop Speak English No prior involvement in research
Inclusion criteria for researcher-participants & practitioner-participants	Working in research priority topic area Interested in the workshop and collaborative research Available to attend Speak English (for workshop 2 we considered including translation but the practicalities of this for an online interactive workshop became too complex)			

Public-participants were contacted by email or phone by a researcher to arrange an online informed consent meeting. Information about the study was emailed in advance of meeting. At the consent meeting potential participants had an opportunity to ask questions about the study and workshop, and verbal or written recorded consent was obtained. Researchers and practitioners were approached by email and sent information on the study. For WS1 researchers were asked to sign and return a consent form via email. For workshops 2–4, to ensure researcher-participants were treated the same as public ones, each researcher/practitioner was met with to explain the study and answer any questions before asking for the consent form to be completed, signed and returned by email.

### Phase 2: data collection

Evaluation data was collected at each workshop using participatory observation, with fieldnotes being typed during and immediately after the workshop. Observations were informed by an observational guide (Additional file 1). Observational notes were completed by at least two C2C team members for each workshop to strengthen validity of the data. The purpose of the observational data was to provide data on behaviours, activities, workshop atmosphere and relational interactions which may be lost to recall or reported differently during follow-up interviews. Qualitative one-to-one interviews with public, practitioner and researcher-participants were semi-structured and guided by a topic guide (Additional file 1). The purpose of interview data was to explore experiences and perceptions of the workshop—including roles, power dynamics and levels and types of engagement. Interview and observational data were triangulated with data created during the workshop itself, through workshop packs, written notes in online whiteboards, or the chatlog function and in WS1 a short survey after the event.

### Analysis

All the data was imported into NVivo 12 software. AB designed the initial deductive coding framework to be used in NVivo. Working with deductive and inductive (NVivo) coding, AM coded all the data, with 33% of data double coded by MF. JS further went through all transcripts to anonymise them for sharing within the data repository, identifying any further themes as they arose through this process. All the data for each workshop was coded in turn to facilitate building a cluster of codes for each workshop. For each workshop, the types of data were coded in the same sequence: first interview data, then chatlog data, finally observation fieldnote data. Codes were analysed within each workshop before being compared across workshops. Codes were organised into themes and overarching themes to answer our research questions. We were interested to identify disconfirming accounts within workshops as well as across workshops. We were also interested to identify different accounts between researcher, practitioner and public-participants – particularly around issues of power dynamics and perceptions of whether participants were able to make contributions regardless of their background or public versus researcher status.

## Results

Table 4 shows the number of participants who expressed an initial interest in each workshop, the number who completed informed consent, and the number who attended the workshop. We did not routinely collect socio-demographic information on participants.

**Table 4:** Recruitment: number of participants contacted, recruited and attended workshops, and interviewed

	Number of public-participant expressions of interest	Number of informed consent processes completed with public-participants	Number of public-participants consented and attending workshop	Number of practitioner-participants (pract) consented and attending workshop	Number of researchers consented and attending workshop (res)	Total participants attending workshop	Total participants interviewed
WS1 Young people	16	14	11	1	4	**16**	4 (2 public, 1 res, 1 pract)
WS2 Somali community	12	12	12	2	4	**18**	4 (2 public, 1 res, 1 pract)
WS3* Air pollution	37	26	21	2	3	**26**	7 (6 public, 1 res)
WS4 Health data	25	10	10	0	4	**14**	6 (4 public, 1 res)
**Total**	**90**	**62**	**54**	**5**	**15**	**74**	**21**

Totals in bold

*For workshop 3 two shorter workshops were held with participants attending either a morning workshop or an evening workshop to enable greater participation. This decision aimed to provide two time slots to increase recruitment amongst a demographically unknown geographical population

Table 5 gives a summary of each workshop content and format.

**Table 5:** Workshop design and content

Workshop 1 (WS1)	*Date and duration:* Saturday 6th February 2021, 3 h *Framing/workshop style:* Game show: points were kept by participants; games were not aimed at skill/technique but building connections. Moments of reflection in the chat were included throughout. One main facilitator, supported by a second *Opener/warm-up:* Measuring the potato brought by participants (smallest and largest potato lose); Zoom fetch including an object that is ‘a comfort’. Following a screen break, a quick movement game where only 3 people could stand at any time *Relationship building exercises:* Creating connections with all participants turning cameras off and turning them back on if a statement was true for them (only showing all those that had that thing in common). Switching roles, with young people asking researchers questions in small groups *Research discussion: *Zoom polls asking questions about the pandemic and school support. Anonymous chat responses to a series of questions around how it feels to be sent out of class, the effectiveness of school discipline, and what participants wished school staff knew more about. Small group task to come up with a new strategy to support student wellbeing at school, which was then pitched to the main group with a vote at the end *Ending*: All participants hiding under a blanket on screen with the challenge to not be the first or last to remove your blanket
Workshop 2 (WS2)	*Date and duration:* Thursday 11 March 2021, 2 h including pizza delivery towards end *Framing/workshop style:* A participatory experience with games, conversation and spoken word poetry. Two facilitators (one British Somali who would be able to facilitate culturally appropriate discussion) *Opener/warm-up:* Welcome and stretches. Introductions and each share one interesting thing and a thing discovered during lockdown *Relationship building exercises:* Checking in with mood through reflection exercise and sharing reflections on things that are good and challenging. Small group game to find the most interesting thing you have in common. Zoom fetch game, including finding an object of hope *Research discussion:* Facilitated discussion on participants experiences, such as their roles as women in the community, what would make it easier to reach out, and quick meal tips *Ending:* Three minutes to free write. Reflection on connections made and checking back in on mood with reflections on experience. Mention of seed funds available with further follow-up emails including discussions about this
Workshop 3 (WS3)	*Date and duration:* Wednesday 21st April 2021 (1.5 h, one morning, one evening workshop) *Framing/workshop style:* Conversation guided by facilitator and illustrated booklet provided to participants before the workshop. One main facilitator with co-facilitators for breakout rooms *Opener/warm-up:* Hello and welcome, session housekeeping. Researcher introductions *Relationship building exercises:* To bring and discuss an object to the screen that represented the workshop topic—air pollution *Research discussion:* Break out room discussions, supported by illustrations in workshop packs. Discussion exercises included ‘in my neighbourhood I want to change…’ and ‘use the key to map air pollution issues in your area’ *Ending:* What could £2000 fund as a community project exploring air pollution
Workshop 4 (WS4)	*Date and duration: Thursday 15th July 2021 (3 h in the afternoon)* *Framing/workshop style:* Game Show: series of small group games that became more elaborate, with points awarded to the teams after each game and a small prize on offer to the winning team. Two facilitators *Opener/warm-up:* A number generator to put people into teams and a game to find the most interesting thing each team had in common. Zoom fetch with an object related to song lyrics (with the link explained by each participant for points) *Relationship building exercises:* Sharing talents and tips (in pairs) and a group challenge to steal the Mona Lisa using the group’s specific talents *Research discussion:* Game that asked participants to decide in a fictional situation whether they would share their health data. Team conversation and writing of a manifesto on health research (shared back with main group) *Ending:* Researcher introductions. Introduction to seed funds available. Discussion about future session

We present the results thematically, making comparisons across workshop case studies in terms of the following over-arching themes, and demonstrating thematic claims with verbatim data:

1. Setting up the space and introductions
2. Conversational dynamics, contributions, roles and identity
3. Impacts of workshops

To denote who is speaking, all participants are labelled according to workshop number. Practitioners have an additional (-practitioner) after their interviewee participant number, and researchers have an additional (-researcher) after their interviewee participant number. A GRIPP2 short form [20] is included as Additional file 2 to illustrate how we have reported on our experiences of public involvement throughout this article.

### Setting up the space and introductions

#### Introductory games

All workshops used a variety of introductory games to begin. In WS1 the game show atmosphere (go and fetch, measuring a potato, awarding points) helped put some young public-participants at ease, moving from “awkward” towards feeling “calm” and able to connect and “bind” (WS1A); “getting us pumped up, excited and ready for something new” (WS1A). In WS2, which had two artist facilitators, the game ‘go and fetch’ (an online version of show and tell) was seen by some participants as significant in building relationships: “it really helps people see the humanity between each other” (WS2A-practitioner). However, some researchers in WS2 found the introductory games did not fit so well, but appreciated the second facilitator, a British Somali poet, who led the group discussions. In WS3 participants were asked to fetch and bring an object to the screen that represented the workshop topic—air pollution. One public contributor who usually lacks confidence to share their views, found this very enabling.

“Making sure that everyone had spoken at the beginning definitely helped, because once you’ve done it once it’s very easy to then do it again” (WS3E)

Within WS4 one participant described how a game had the right balance of fun and engagement, another said there was a “level playing field and everyone collaborating and being able to make a contribution” (WS4A). In contrast, two WS4 researcher-participants felt that despite the games helping to break down barriers, the games went on for too long.

“I think we had too many activities in a way, where we were getting to know each other and helping to build that participatory collaborative work that we were doing… it felt that we ran out of time to actually discuss the really important thing…” (WS4D-Researcher).

Generally, researchers’ views were more mixed about the games, with some experiencing new and creative ways of engaging with people which they said that they would adopt in their own future practice. Other researchers were more familiar with some of the techniques used, and some were unsure of the value or benefit of the extended time given to this. In contrast, almost all public-participants reported enjoying and appreciating the introductory games and activities. When asked to describe the atmosphere in the workshop, public-participants used only positive descriptions such as: engaging, enjoyable, interesting, great fun (WS1); comfortable, safe, exciting, uplifting, enlightening (WS2); familiar, comfortable, non-judgemental, open, enjoyable, equitable, engaged, interesting (WS3); welcoming, friendly, equitable, fun, level playing field, wacky, quirky, open, relaxed, fun, light, friendly, slightly competitive, gamey, enjoyable (WS4). There were varying perspectives on the extent to which the activities were seen as creative, with some researcher-participants having expectations of a more hands-on creative activity, whilst acknowledging the limitations of Zoom and social distancing, which was operating during the workshops.

### Introducing people and roles

The workshops took a variety of approaches to introducing people, attempting to change the usual structure where researchers are introduced through their role and research interests:

“We did introductions for a fair while but it wasn’t focused on what people did. Obviously, there was a bit of like ‘I’m a researcher at so and so University or I volunteer or whatever it was, but there wasn’t a focus on that, which I think equalised everyone as just people interested in that conversation rather than we must listen to that person because they are a learning person, do you see what I mean? It felt it equalised very quickly.” (WS2-Practitioner)

Researchers had various responses to this. Some had expected that they would have an opportunity to speak about their work:

“I was expecting it at the beginning if I’m honest during the introductions. I was almost expecting the people attending from a research background to almost give a very short summary of their research and what they’ve been involved in” (WS2C-Researcher)

Another suggested additional guidance would have been helpful, which was developed as workshops progressed:

“I think I was quite a bit confused in my role…. I sort of outed myself and said, ‘I’m also a researcher,’ but I was then meant to be a participant, so I was a bit confused… It just meant the workshop started with me—a bit unsure about if I’d done something right or wrong, and what my role was in the workshop…. I feel like we went… we went into it really quickly without setting ground rules or knowing what was going on.” (WS3C-Researcher)

This was changed by WS4 as we learnt to give researchers more guidance as to how the workshop would run, and that role introductions would not happen at the beginning. Whilst there were no role introductions in WS4, one researcher thought that public-participants still “knew who the experts were” (WS4D-Researcher). However, three WS4 public-participants connected not knowing who the researchers were to a feeling of equitability:

“not knowing who the researchers were in the beginning until later meant that it felt very equitable” (WS4A).

“I was actually surprised at who were the specialists in the room. The people that were being researchers I didn’t think they were the researchers so when they revealed themselves…I was like, ‘wait, you’re the researcher, okay’. It surprised me that they were able to blend in with the participants... Everyone then felt more equal within the room so more comfortable to say this is what I’m thinking without the judgement.” (WS4B)

### Conversational dynamics, contributions, roles and identity

In WS1 young participants described feeling listened to, felt their views mattered, that their “voice was heard”, that “everyone’s opinions were heard” (WS1-survey). Young people particularly valued an anonymous chatlog discussion: “games where you were anonymous- I feel people opened up most” (WS1-survey). The anonymous chatlog contributions were to have an important influence on what happened after the workshop, due to the power of young people’s words, particularly around the impact of school discipline on wellbeing. However, whilst a researcher-participant and practitioner-participant in WS1 enjoyed the anonymous chatlog, they both shared a concern to ensure that with any anonymous contribution there was a way to follow up in case a safeguarding issue was identified.

In WS3, participants were posted an illustrated workbook created by the workshop facilitator. This workbook was integral to the workshop and participants were invited to read and complete it before the workshop if they wanted to. One participant whose first language was not English and who knew little on the topic of air pollution found the workbook:

“Was really easy explained and gave me the possibility, as I didn’t have any knowledge about it [the topic], to get involved and feel involved in the workshop… I knew what was gonna be next, and if I didn’t understand something, I was looking at it” (WS3A).

In WS3 there was a shared sense amongst public-participants that everyone could contribute on a similar level.

“It felt like it was everybody involved and everybody got a chance to say what they wanted, not being directed too much.” (WS3G)

Two C2C team observers interpreted dynamics where researcher-participants spoke first and public-participants were then reticent to speak, suggesting an unequal dynamic: “It did still feel a bit like an “us and them” meeting.” (WS3-observational fieldnotes). However, public-participants framed differences in contribution as unproblematic, even positive, because it indicated there was no pressure to speak if you didn’t want to.

“I think it’s nice because when you build a space that is safe, then it’s still their own choice… there was no pressure, so I think this is nice, in a way, that somebody that doesn’t feel comfortable to talk doesn’t have to talk, but then others take part” (WS3A).

This contradiction highlights that impartial workshop observers can only interpret how others are experiencing a workshop; these interpretations may not always be aligned with participants’ own experiences.

In WS4 a public-participant experienced the workshop as challenging normal dynamics:

“In other settings and at work you find that it’s the usual suspects talking…you don’t necessarily hear from everyone whereas this experience was different to that. It felt like everyone was contributing and it wasn’t just the same people talking all the time.” (WS4A).

In contrast, researchers tended to find it more difficult to know when to contribute to conversations, and how to add in their related knowledge. Some felt a need to “sit back” from making contributions, as different researchers from WS2 and WS3 both expressed:

“I knew information that could have maybe helped that conversation, but I didn’t say anything ‘cause I didn’t want to be, ‘Oh, I’m the expert, by the way.’(WS3C-Researcher).

“I didn’t want to turn around and say ‘I’ve studied this’ or ‘I’ve researched this’ when people were talking openly about personal experiences. I think sometimes I found it quite hard to know when to jump in” (WS2C-Researcher).

A WS2 practitioner-participant felt researchers were right to ‘tread lightly’ initially but recommended bringing in researcher-participants more towards the end:

“when they [Somali women] have someone from not their culture coming in and telling them, so it is kind of [important to] tread lightly….I think they [researchers] got it right… towards the end, you know, they [had] already won over the ladies, … they could have maybe shared a little bit more about their background and what they’ve done, because towards the end everyone was comfortable… the conversation was flowing” (WS2D-practitioner).

Overall, most researchers felt their contribution had been more limited than they expected:

“I mean I was able to deliver a short pitch and I think that was a reasonable contribution but, as I said, I think it was a little bit rushed at the end and I felt like perhaps I had a little bit more understanding and subject matter knowledge than I was able to provide and probably part of that was not wanting to lead people particularly in a direction.” (WS4C-researcher)

Public-participants’ perspectives on this varied. Public-participants described flow and equity, but they also wanted researcher expertise. One said it would have been good to hear about researchers’ contributions at the beginning. Another shared the sense of a missed opportunity to speak more with researchers, whilst also having benefitted from not knowing who they were in the first half of the workshop:

“I really enjoyed the last part where we were able to ask specific questions of the researchers… but then I also enjoyed the fact that we didn’t know who the researchers were, I thought that was really well done in that it was held back otherwise we may have been slightly reliant on those individuals in our different rooms to maybe lead the discussion and that wouldn’t have been a positive thing I don’t think. I would have perhaps liked a little bit more interaction with the researchers but how do you do that? There’s just not really enough time I suppose....” (WS4E).

In contrast to researchers’ eagerness to voice and share their identities and work, one WS3 public-participant particularly kept quiet about their own identity. This participant did self-censor their verbal contributions as they felt “vulnerable” about expressing their opinion, as part of a marginalised group. However they still felt part of it because they could write in the workbook and see everyone. This public-participant suggested that anonymising contributions could have been helpful, just as the artist partners had done in WS1, so nobody could tell who said what: “… even a little box to put in an anonymous comment…basically for minorities or people who just might not feel comfortable saying something” (WS3F).

Finally, data suggested the different roles that people brought to the workshops, typically determined by whether they were employed or not within the topic area. For some researcher-participants, this went hand in hand with some unclarity around what their own contributions could be:

“Was I participating in the discussions as an academic, or as a member of the public?” (WS3C-researcher)

One researcher-participant noted that the workshop felt ‘participant-driven’ in a way that was new to them:

“It was a completely different atmosphere, very much participant-driven rather than perhaps what I’ve been involved in before.” (WS2C-researcher)

Whereas a practitioner-participant felt familiar with the workshop structure:

“I work with commissioners and service providers but I also work with people with lived experience and communities on the ground, so I guess I’m in the middle all the time, so I just kind of learnt that everyone is a person with a view and just have to kind of—your view is as valid as the next person’s.” (WS2A-practitioner)

### Impacts of workshop

#### Impacts on workshop participants and connections

Within WS1 one young person, when asked later about their role and contribution in the workshop, described stepping into a leadership role:

“Like I kind of took a leader role on that kind of [discussion]... So, kind of a leader role of just expressing my opinion and saying how I wanted it [school environment] to also be changed as well.” (WS1A)

In WS2 one participant felt empowered and informed to broach the topic of mental health with their father for the first time:

“It has prompted me to have conversations with other people, namely my Dad, because my Dad views mental health as a taboo, but having listened to most of the women speak [in the workshop], it opened up the floor for me to have a conversation with my Dad… I was kind of challenging him on that, and I feel like I’ve gained a lot of knowledge, myself”. (WS2B)

Similarly, a WS3 public-participant felt more equipped to begin conversations with friends on the topic of air pollution. A WS2 community practitioner found that the techniques used in the workshop, helped her reflect and develop her own practice:

"It was really interest[ing] to me to see a different way of working because we do a lot of focus groups ourselves and engagement events… it’s really interesting to hear a different way of asking questions… we’re taking all the learnings… do a storytelling workshop ourselves for community groups" (WS2A-practitioner)

These examples fit with the idea of the co-creation of emergent knowledge, which may lead to lasting personal knowledge [5]. In WS4 a public contributor saw how the creative play facilitated discussion on research topics:

“You’ve created something together and even if the thing you’ve created isn’t as an object but a story about stealing a painting, that’s still something you’ve made together as a group…creating something together bonds a group in a way I think that nothing else can… in those games, already having talked about all your super powers, now we’ve all shared our talents; you‘ve shared your talents so we’ve already begun, we’ve already played talking about ourselves – ‘oh this is a bit of me that I’ve shown you’, so we’ve already done it so then showing you something else about me [is] less scary” (WS4E).

However, in contrast, a WS4 researcher-participant felt the workshop hadn’t “really nailed the subject matter” (WS4C). This researcher-participant was hesitant about participating in a similar workshop format again, and the online nature meant that there had been less opportunity to follow up connections.

“There were loads of interesting people in the workshop who had really interesting insights, who I would have loved to go and speak to a bit more in depth about various issues or thoughts or perspectives that they had, and there wasn’t an opportunity to do that” (WS4C-Researcher).

Some public-participants also fed back that they would like to swap contacts with both researcher-participants and other participants, to build on the workshop discussions and connections, which was not always possible in the time allotted for the workshops. In addition, online workshops made it difficult for this to happen organically, as they might within an in-person workshop during coffee breaks.

### Further developments after workshops

At the end of the workshops there were varying amounts of time to discuss next steps, potential collaborative projects and how these could be supported by seed funds. Some participants had expected or wanted something more from the workshop towards the end:

“Having feedback on that idea to see if it’s actually do-able or not, or giving us more support or more continuity and follow-up” (WS3A).

Whilst this was offered via email contact, which some participants took up, on different workshops there was limited project staff time to take this on. The available £2,000 seed fund was aimed at supporting an ongoing conversation and development towards a research project in an equitable way. Indeed, part of the rationale for having two of the workshops linked to ARC West research priority setting, was the intention that workshop discussions could feed into research projects developed with the resources of ARC West. This worked well with WS1, as young people’s anonymous contributions about their experiences of school discipline, when discussed at the research priority setting group was influential in setting up a project on the impact of school discipline on pupil wellbeing [21]. The young people involved in the workshop were invited to be part of a pre-existing Young People’s Advisory Group and to train up as peer researchers, who then went onto interview other young people about their experiences of school discipline. With WS3 the air pollution conversations were harder to fit and public health departments who were also members of the research priority-setting groups established other priorities they wanted to develop as research. Whilst one C2C team researcher had various conversations with public-participants who had ideas for the seed funds, unfortunately nothing came to fruition. Discussions in WS2 and WS4 included suggestions that follow-on events were needed with researchers playing a more active role in developing research priorities from these discussions. Further meetings followed WS2 bringing together the community partner and two public-participants to develop follow-on training sessions for the community. Due to staffing changes, this was not able to progress, but further relationships between the university and community organisations have been maintained, serendipitously, through other health projects. WS4 participants expressed interest in being involved in further work on health data, but without capacity amongst attending researcher-participants to lead further discussions related to their focus areas, no subsequent projects occurred.

## Discussion

Overall, the study was able to successfully engage members of the public and local communities and researchers in health research workshops. Our data presents a complex picture about whether the creative processes facilitated the development of more equal/neutral interpersonal relationships between researchers and public-participants and supported the deconstruction of power hierarchies. How do we make sense of conflicting accounts between public-participants and some researcher-participants, with the latter being more likely to be critical of the process of the workshops than the public?

To aid our analysis of this, it is useful to frame our results within different dimensions of power. Power can be visible and seen, hidden through agenda setting and decision-making processes, and invisible through usual practice, values and assumptions [22–25]. These different dimensions of power have been used to help understand power dynamics within co-production processes [26, 27] and we illustrate how different dynamics were present within the workshops (Table 6). The sections below cover these visible, hidden and invisible power dynamics in more depth, alongside a reflection on the challenges we faced and recommendations we suggest as a result of these (Table 7).

**Table 6:** Power dynamics within C2C workshops

Dimensions of power	Description	Workshop structure and dynamics present
Visible	Institutions, hierarchies and roles, observable conflict and disagreement, resources	We attempted to make researcher roles less visible through not introducing researchers as such at the beginning of some workshops, although this created some discomfort and confusion for some of the researcher-participants We tried to link resources to post-workshop activities, to enable development of future collaborative work. However, this had mixed success
Hidden	Agenda-setting and decision-making processes	Initial themes came from different discussions including community organisations, young people, cross-sector priority setting groups, and University research themes. Public-participants reported an open and engaging conversation Our initial focus on activities attempted to move away from traditional meeting agenda approaches controlled by researchers. This worked well from public-participants’ perspectives, although less well from researcher perspectives
Invisible	Usual practice, language, beliefs, attitudes, values and assumptions	Assumptions of how things would work were sometimes disrupted by artists and facilitators Researchers sometimes assumed that their research interests would be given airtime, but this was often less than they had expected or hoped for Researchers and public-participants tended to value different aspects of the workshops

**Table 7:** Challenges and Recommendations

Challenge	Recommendation
1. Expectation setting	Spend as much time as possible clarifying the aims and outcomes of a workshop, e.g. whether a workshop is primarily a space for knowledge exchange, research prioritisation, or setting up of a new research project, etc. These aims will influence participants’ expectations and support them in forming ideas about what their roles and contributions could be
2. Roles in workshop: are you employed to be there or not?	Participants are likely to turn up with different agendas, expectations, and ideas about the use of their free/employed time. Creative activities and an external facilitator can be valuable in moving away from workplace agendas and creating space to explore new ideas
3. Bringing community needs into research	Within higher education, translating community-driven ideas into funded research projects faces capacity challenges, particularly relating to funding and researcher time. This type of creative workshop lends itself well to research prioritisation around themes and may be most appropriately used in (1) informing future funding bids, (2) research prioritisation around themes at the start of new funding
4. Support available to researchers aiming to pursue co-produced research projects is limited	Researchers are time-poor and work put into developing community relationships is not typically rewarded within the career pathways of academia. Co-production could be further supported by increasing researcher access to training, resources, networks and funds. Evolving and sometimes contradictory terminology (for example see debates between [33, 34] is also a challenge (participatory; co-produced; co-created; collaborative) which training and increased interdisciplinary working might reduce
5. Making space for productive discomfort	Collaboration and relationship building can be a time-intensive process that requires building trust and removing barriers to equitable participation. To some, this process may feel like losing some power or agency. Managing expectations of all participants is important
6. Equality between co-production participants is not visibly self-evident	Not every participant in a workshop will contribute the same way or amount. Some may prefer to speak less than others. Confident facilitation can encourage participants to contribute in a way that suits them
7. Setting up a safe respectful discussion space—the value of a skilled facilitator	Revealing identities or lived experience may cost some participants more than others. Researchers involved are likely to feel more comfortable discussing a topic, while public-participants may require more facilitated support to engage
8. Co-production needs to work for everyone	Deconstructing power dynamics can be an important process in co-produced research. It’s important that researchers do experience agency in the process of building research projects or knowledge exchange. However, this should not come at the expense of public contributors’ ability to set the agenda. A clear process and training around different approaches to co-production are likely to be beneficial
9. Developing a sense of ownership	Deconstructing power dynamics can leave participants without a sense of ownership when it comes to taking ideas forwards. This links to recommendation 8; further facilitation is then required to support reconstruction of new roles in emerging follow-on projects
10. Further dedicated staff time needed to pursue follow on projects	Build in staff time to follow up ideas and support further collaboration. Make use of research support staff such as public involvement and engagement professionals who can balance and bring together different ideas and agendas, and have the time to set up further workshops When working online, pay attention to creating space for informal relationship building (and potential exchanging of contact details) which is likely to happen organically at in-person events

### Visible dimensions of power: roles and conversational dynamics

Our experimentation with not introducing researcher-participants as researchers at the beginning of a workshop produced some unease and discomfort, with a sense of researchers not knowing the rules or how to interact. We took from this a need to explain our intentions more clearly before each workshop (Table 7, point 1), and this was something we aimed to do in later workshops. Some of these tensions were still present in WS4, with some public-participants articulating that they would appreciate a clearer contribution from researchers because they wanted to be able to contribute usefully to the research topics. Due to their different employment status, public and researcher-participants may arrive at meetings with different agendas, expectations, and ideas about the use of their free/employed time. Research time is highly pressurised, with the system favouring quantity over quality, at the expense of creativity [28], which may allow researchers precious little time to dedicate to open exploration of new ways of working (Table 7, point 2). Despite offering a small seed fund for follow-on work, it was clear that maintaining momentum for research prioritisation would require dedicated staff time, most likely by research support staff (e.g. PPI/public engagement professionals) working in collaboration with both public-participants and researchers. We found capacity challenges limited our ability to provide support for follow-on work (Table 7, points 3 and 4).

We debated as a team about our findings in relation to the position, experiences and views of researcher-participants in the context of the positive experiences reported by our public-participants. Given that our aim was to disrupt established power positions, some of the team considered some feeling of discomfort on the part of researcher-participants as not necessarily inappropriate. However, we also felt that just because someone felt uncomfortable, or unclear in their role, that did not necessarily mean that we were being successful in dismantling power dynamics. Yet, there is an identified dynamic within co-production where professionals “engage in a process which reduces their professional power, with the experience of challenge and discomfort being a key feature of that process” [29]. The workshop structure meant that researchers’ roles were partially deconstructed, however, we did not have the space or guidance to help reconstruct these roles into something different, which meant that roles were not clear. This may have contributed to researchers getting less involved after the workshops. More broadly, there are important epistemological, institutional and personal challenges that can occur within the development of co-produced research [30, 31]. Epistemological challenges include how different forms of knowledge and experience are integrated, institutional challenges include issues such as institutional hierarchies of knowledge, alongside short-term project based funding, workforce casualisation and work intensification [32], and personal challenges include the navigation of these pressures at individual and system levels. A key learning from the project is that it is important to support researchers within the process of dismantling existing power positions, so that any discomfort can be productive and experienced as an important part of the co-production process (Table 7, point 5). Additionally, the challenges and recommendations in Table 7 arise from reflections to support meaningful research engagement, which can be constrained by institutional priorities and challenges that are likely outside of the control of those attempting to support the type of social engagement practice we discuss here.

### Hidden dimensions of power: equality between participants, identities and agenda setting

Equality between co-production participants is not visibly self-evident [19]. In some ways our research question was difficult to answer because there are not clear visible markers about what constitutes equal participation. Evidence of this can be seen through different public and researcher- participants’ perspectives about what constituted equal contribution, with C2C team observers wondering whether conversations were being dominated by a few participants, whereas some public-participants reported being at ease with how and when they wanted to contribute, some preferring to speak less, yet being comfortable to contribute when they wanted. Thinking around equal contribution therefore should be seen in the context of participants’ experiences (and reports) of their ability to contribute, rather than only from an observational perspective (Table 7, point 6).

Another dynamic around visibility and invisibility was how different people felt about voicing their identities. Whilst researchers were keen to voice their identities at an early stage in the workshop process, one public-participant from an invisible minority saw the potential costs of voicing identities especially where stereotypes and social inequalities were apparent within conversational dynamics. It may cost some people more than others to make their identities visible. This may be more likely when covering a broad public health topic as participants may be from very different backgrounds but affected by the same health landscape. Similarly, this situation may arise when sharing lived experience around a potentially stigmatised health issue. We found great value in external facilitation, particularly when the workshop lead was confident in setting up a respectful and safe environment from the start of a session. This is particularly important when focusing on potentially sensitive health topics (Table 7, point 7).

The scope for participation can be narrowed by more powerful actors who are better resourced to design participatory processes and shape the social space in line with their own needs [19, 35]. We tried to disrupt this dynamic by giving space to creative facilitators to design the workshops. However, researcher-participants often felt that there was not enough time for them to share their research interests and topics. Part of this may be a result of having to shorten the workshops to account for their online nature, but different public and researcher-participants priorities are evident in quotes like “it felt that we ran out of time to actually discuss the really important thing …” (WS4D-Researcher). If we return to the definition of the creative process, engaging in “meaningful actions and interactions” [11], in some ways the initial activities may have been less meaningful to researcher-participants, with less experience of their own agency. Perhaps because researcher-participants did not own the agendas of the workshops, the study team found it more difficult to involve researcher-participants in post-workshop discussions, as they did not own the project, and sometimes the workshops had not sufficiently met their needs. If the desired aim is to create more co-produced research that is done 'with' the community, researcher-participants also need to feel and experience a sense of agency to facilitate partnerships with public-participants to create change. Co-production has to work for everyone (Table 7, point 8). These points relate well to wider structural barriers to engaged research that have been highlighted, including processes of casualisation of the academic workforce, work intensification and projectification [32]. Only in WS1 was a full research project generated from discussions, as part of a wider piece of work with the ARC West Young People’s Advisory Group, which new members joined from the workshop [21]. Key enablers for this work were a well-established and supported Young People’s Advisory Group, alongside passion from senior leaders for young people’s voices to be heard through ARC West’s research prioritisation processes. This successful outcome in one out of four workshops highlights the challenge institutional structures, and their changing priorities over time, present to engagement projects such as this. The main project aim was to examine how creative approaches could help develop relationships. In aligning with existing themes and priorities we hoped that some of the workshops would result in new research projects (however this was less within our control)—whilst only one workshop did, the other three did instigate conversations and connections, which are harder to quantify as outcomes.

### Invisible dimensions of power: usual practice and values

Co-production cases have illustrated “how the ability of elites to step back was central to allowing the renegotiation of roles and responsibilities of participants” [36]. Researcher-participants when interviewed similarly used the phrase “sit back” to ensure that public-participants had their say. Researcher-participants also felt less at ease with when and how to contribute their knowledge. Changes in dynamics and feeling less in control of a process than in usual practice can “require vulnerability among all stakeholders” [4]. More broadly there are debates about how and when to merge experiential and evidence perspectives. Through the strategies outlined in Table 2 Rationales for workshop design we attempted to address some of the power dimensions of co-production, disrupting ways in which researchers are seen “as holders of knowledge” and public-participants “as receivers of scientific expertise” [19]. Whilst some dynamics were disrupted in the moment of workshops, longer term impact of this was not facilitated. Whilst we attempted to create a level playing field within our workshops, enabling a “space to talk” where there was a shared dialogue [37]; these spaces were effectively “bubbles of co-production” [38]. “Space to change” [37] after the workshops to adapt in response to workshop dialogue was harder to hold, due to the limited resources to continue conversations, with less sense of ownership from researcher-participants to run with and develop conversations further in some cases (Table 7, point 9). Limited workshop time (due to running the workshops online) may also have been a factor. Others who have tried similar approaches and had more success with follow-on projects conducted their workshops over a period of two days in a face-to-face setting which may have been more conducive to relationship building [3]. For effective longer-term co-production, shorter online creative workshops may require ongoing facilitation, and multiple sessions with the same group, to produce the same results as a single, longer face-to-face event, as originally planned (Table 7, point 10).

### Strengths and limitations of the research

The nature of our team was multi-disciplinary (health sociologist, anthropologist, public engagement expert, participatory performance and impact researcher) which provided diverse and sometimes conflicting perspectives, which have informed the write-up of this paper. The project took place within a medical school which has a strong leaning to scientific expertise and authority. Health research governance processes created restrictions and requirements that arts researchers and practitioners may not be familiar with, or that are not in line with their own practices, and it was difficult to make space to challenge this within the project. Through our different workshops we have explored how different voices that sometimes contradict and conflict can “be voiced and included in a legitimate way”[19]. This has also been a puzzle and a challenge for the writing of this paper to include different perspectives and experiences of the same workshop process. It has been highlighted that in trying to shift power dynamics within co-production, “conflicts are unavoidable, and it is paramount to resist their premature closure” and that there is “creative and productive importance of contestation, pluralism, informed dissent and difference” [19]. By contesting each other’s knowledge claims, we have tried to be diligent and thorough, and considered all perspectives in writing this article. In working to hold multiple perspectives simultaneously and in conversation with each other, our process mirrored aspects of parallaxic praxis [39], which is a research method that emphasises the value of combining different experiences, perspectives and approaches to meaning-making. Through combining perspectives from different participant groups, the interdisciplinary research team and our collaborators, our project brought together multiple experiences and approaches to meaning making.

We have tried to report our complex findings with a degree of humility in where we went wrong, could have done things differently and where others have been critical of the workshops, coming to terms with our own “ragged fringes of human understanding—the unknown, the uncertain, the ambiguous, and the uncontrollable” [36]. We set out to experiment with different structures and workshop processes; not all of these were seen as successful to all participants, but they did disrupt and challenge some established practices and power systems. Others in this field have also highlighted that such creative methods are not without their challenges [7].

COVID-19 restrictions and social distancing meant that we were hampered in our original aims of doing more practical, hands-on creative tasks or “making activities” [7] in a face-to-face environment. However, one of the legacies of COVID-19 lockdown practice is that a lot more public involvement and engagement still takes place online, as it can be more accessible to some populations (but consistently also entails digital exclusion problems) [40]. Our findings are particularly relevant to these ongoing online practices.

## Conclusions

Overall, our findings align with those of Phillips et al. [5] in that the complexities of arts-based co-production can be seen in terms of tensions, particularly between cultivating a collaborative, creative process within our workshops and the subsequent development of specific research projects [5]. Our attempts to facilitate more equal relations were impacted by wider structural biases and processes [41]. Our results highlight that whilst deconstructing hierarchies is one element in changing power dynamics, the importance of reconstructing new roles needs to be considered in more practical terms for longer term, collaborative relationships to develop. Whilst creative methods can facilitate emergent processes and level some power relations in the moment, we found that these “bubbles” of co-production [38] could pop and disintegrate in the longer term, with more difficulties in creating longer-term material outputs [5]. Engaged research projects are situated within academic structural systems with barriers that are not easily resolved by researchers within specific research projects [32].

### Supplementary Information

**Additional file 1.** Observation template and interview topic guide.

**Additional file 2.** GRIPP 2 Short form.

## Data Availability

The datasets used and analysed during the current study will be available to bona fide researchers from the University of Bristol data repository upon request subject to a data access agreement following Wellcome Trust guidance https://data.bris.ac.uk/data/.

## Abbreviations

C2C: Create to Collaborate
COVID-19: Coronavirus disease 2019
EBI: Elizabeth Blackwell institute
KWMC: Knowle west media centre
NIHR ARC: West National Institute for Health Research Applied Research Collaboration West
WS1: Workshop 1
WS2: Workshop 2
WS3: Workshop 3
WS4: Workshop 4
WS5: Workshop 5
