# Supplementary material for: Create to Collaborate: using creative activity and participatory performance in online workshops to build collaborative research relationships
Source: Res Involv Engagem. 2023 Dec 6;9:111. doi: 10.1186/s40900-023-00512-8 (PMC10701968; doi:10.1186/s40900-023-00512-8)
Supplement: Supplementary file 1 — Additional file 1. Observation template and interview topic guide. [file 40900_2023_512_MOESM1_ESM.docx]

Additional file 1 Observation guide and interview topic guide

Create to Collaborate Observation Guide

1. **Process: Describe the group dynamic:**
2. How are participants engaging in the activities?
3. Do the creative activities help to get everyone involved, and if so how?
4. How are participants engaging in the discussion?
5. What kinds of contributions are participants making?
6. How are people working together?
7. Do some people take the lead/ more engaged/ vocal?
8. Are participants contributing at a similar level?
9. Any responses or instances that stand out?
10. How are people’s perspectives, knowledge and skills valued and incorporated?
11. Does the group dynamic change and if so how?

**2) Example workshop content questions for Workshop 3 (air pollution)**

a) What objects do people bring to visualise air pollution and why?

b) What are people’s concerns in association with air pollution?

c) What kinds of changes do members of the public think councils should make in relation to air pollution?

## Interview topic guide for participants after C2C workshop

**Protocol States:** interviews are aimed at understanding participants experience of and responses to the workshop and how people perceive themselves as potential collaborators in a research project (including their role and potential agency)

PRE-INTERVIEW SET UP:

- *Thank you for taking part*.
- *Remind participant of consent form (already signed). Explain again about recording protocols, anonymous use of data, free to stop at any point.*
- *Please feel free to ask questions as we go, and to speak openly and honestly. Just let me know if there’s any questions that you would rather not answer.*

**MOTIVATIONS AND POSITIONALITY**

1. Why did you want to take part? [What did you hope to contribute to and gain from the day?]

**EXPECTATIONS**

1. What did you expect coming to the workshop?
2. Have you taken part in these sorts of projects before? [What were they like and what did you contribute to those? How did you collaborate with other people in that project?]

**EXPERIENCES, PERCEPTIONS, INSIGHTS**

1. Describe your workshop experience to me [follow up probes based on narrative]
2. What were the most exciting, fun or interesting parts of the day for you?
   1. Can you describe those moments?
3. Was there anything that surprised you?

**CREATIVE/PLAYFUL ACTIVITY**

1. Was this your first time doing these sort of activities/ games on zoom?
2. How do you feel this activity/ workshop pack helped prepare you for the discussion/ reflective questions?

**CONTRIBUTIONS**

1. What do you feel you contributed to the workshop? (Prompts: activities/ games/discussion)
2. Did you feel as though you were able to contribute in the way that you wanted to? Why/why not?
3. Did you feel more able to contribute more in some parts of the workshop than others? Why was that?
4. How would you describe the roles of the workshop participants? What role you do you feel you took on?

**RELATIONSHIPS**

1. Did you already know anyone at the workshop?
2. Do you feel you built any connections with new people during the workshop? How did that happen?
3. How would you describe the atmosphere during the workshop? Did it change?
4. What do researchers and policy makers need to do differently to make you feel more at home/at ease/comfortable in sharing your ideas?

**EXPERIENCES AFTER THE WORKSHOP**

1. Has anything happened since the workshop? What would you like to happen as a result of the workshop? Please describe
2. Do you have any thoughts on how seed funds could help to support the ideas generated through the workshop?
3. Would you like to carry on being involved in developing research agendas in this way?

- if yes, what role would you like to have?

- If not, do you mind saying why the workshop experience was enough?
