# Supplementary material for: Create to Collaborate: using creative activity and participatory performance in online workshops to build collaborative research relationships
Source: Res Involv Engagem. 2023 Dec 6;9:111. doi: 10.1186/s40900-023-00512-8 (PMC10701968; doi:10.1186/s40900-023-00512-8)
Supplement: Supplementary file 2 — Additional file 2. GRIPP 2 Short form. [file 40900_2023_512_MOESM2_ESM.docx]

# Additional file 2 GRIPP2 SHORT FORM

| **Section and topic** | **Item** | **Reported on page no** |
| --- | --- | --- |
| 1: Aim | Report the aim of PPI in the study | The aims of public involvement are reported in the section Aims and Research Questions on p.4 |
| 2: Methods | Provide a clear description of the methods used for PPI in the study | The methods used for public involvement are reported throughout the methods section, from study design on p.4, including Phase 1: Working with community and artist partners on p.5, rationales for workshop design on p.6, and Table 3 workshop theme, design strategies and inclusion criteria on p.9. |
| 3: Study results | Outcomes—Report the results of PPI in the study, including both positive and negative outcomes | The results of public involvement are reported throughout the results section, including both positive and negative outcomes on p.11-19. |
| 4: Discussion and conclusions | Outcomes—Comment on the extent to which PPI influenced the study overall. Describe positive and negative effects | The extent to which the creative public involvement workshops then influenced continuing research relationships is outlined in Section 3.2 Further developments on p.19 after the workshops and through the Discussion section from p.19-24. |
| 5: Reflections/critical perspective | Comment critically on the study, reflecting on the things that went well and those that did not, so others can learn from this experience | Table 7 on p.21-22 outlines our Challenges and Recommendations. Through the Discussion and Conclusion we critically reflect on our experiences in the light of theoretical notions of power within public involvement. |
